# Anatomic tibial component design can increase tibial coverage and rotational alignment accuracy: a comparison of six contemporary designs

**DOI:** 10.1007/s00167-014-3282-0

**Published:** 2014-09-13

**Authors:** Yifei Dai, Giles R. Scuderi, Jeffrey E. Bischoff, Kim Bertin, Samih Tarabichi, Ashok Rajgopal

**Affiliations:** 1 Zimmer, Inc., P.O. Box 708, Warsaw, IN 46581-0708 USA; 2 Insall Scott Kelly Institute, 210 East 64th Street, New York, NY 10065 USA; 3 723 Mont Clair Drive, North Salt Lake, UT 84054 USA; 4 Burjeel Hospital for Advanced Surgery, Sheikh Zayed Road, P.O. Box 114448, Dubai, United Arab Emirates; 5 Medanta Bone and Joint Institute, Sector 38, Gurgaon, 122002 Haryana Republic of India

**Keywords:** Total knee arthroplasty, Tibia, Fit, Morphology, Compromise, Rotational alignment, Coverage, Overhang

## Abstract

**Purpose:**

The aim of this study was to comprehensively evaluate contemporary tibial component designs against global tibial anatomy. We hypothesized that anatomically designed tibial components offer increased morphological fit to the resected proximal tibia with increased alignment accuracy compared to symmetric and asymmetric designs.

**Methods:**

Using a multi-ethnic bone dataset, six contemporary tibial component designs were investigated, including anatomic, asymmetric, and symmetric design types. Investigations included (1) measurement of component conformity to the resected tibia using a comprehensive set of size and shape metrics; (2) assessment of component coverage on the resected tibia while ensuring clinically acceptable levels of rotation and overhang; and (3) evaluation of the incidence and severity of component downsizing due to adherence to rotational alignment and overhang requirements, and the associated compromise in tibial coverage. Differences in coverage were statistically compared across designs and ethnicities, as well as between placements with or without enforcement of proper rotational alignment.

**Results:**

Compared to non-anatomic designs investigated, the anatomic design exhibited better conformity to resected tibial morphology in size and shape, higher tibial coverage (92 % compared to 85–87 %), more cortical support (posteromedial region), lower incidence of downsizing (3 % compared to 39–60 %), and less compromise of tibial coverage (0.5 % compared to 4–6 %) when enforcing proper rotational alignment.

**Conclusions:**

The anatomic design demonstrated meaningful increase in tibial coverage with accurate rotational alignment compared to symmetric and asymmetric designs, suggesting its potential for less intra-operative compromises and improved performance.

**Level of evidence:**

III.

## Introduction

Though total knee arthroplasty (TKA) is a largely successful procedure [7, 15, 16, 31], malrotation of the prosthetic components has been linked to poor clinical outcomes [1–3, 24, 27]. It has been shown to be a major cause of pain and functional deficit after TKA [2, 3, 24] and lead to over 50 % of painful TKA cases [25]. Therefore, ensuring proper rotation of the tibial component is a key surgical objective during TKA. However, focusing solely on ideal rotational alignment may force compromise on other surgical objectives, including component overhang and tibial coverage. Component overhang has been shown to cause soft tissue irritation or overstuffing of the joint space and associated compromise of range of motion [6, 8, 13], and up to 25 % of the occurrences of persistent knee pain after TKA [23]. Overhang of a properly rotated component is determined by the shape and size of the TKA design; and reducing excessive overhang may entail compromising alignment or size of the component [21], potentially leading to component subsidence and loosening [8] due to compromised cortical support [4].

Several morphological assessments concluded that contemporary tibial component designs do not fit global population equally well [9, 19, 34, 37]. These studies focused on basic dimensions of the proximal tibia, such as anteroposterior (AP) dimension and mediolateral (ML) width, and described the resection shape using aspect ratio [9, 19, 22, 34, 37]. However, these metrics have limited ability in characterizing the asymmetric proximal tibial plateau and global anatomic variations. A recent study showed that ethnic variations in the proximal tibial morphology are dominated by size [11], suggesting that decreased component fit in some populations, especially Asian, is due to limitations in the sizing scheme, rather than shape deficiencies. Similar results were also reported elsewhere, which indicated that differential performance across ethnicities may be attributed to design features in smaller component sizes [14]. Beyond morphometrics, Incavo et al. [20] pioneered the assessment of tibial coverage using digital templating, and conflicting conclusions have since been drawn on whether asymmetric designs offer increased tibial coverage compared to symmetric designs [20, 32, 36], although asymmetry between medial and lateral compartments of the tibia plateau has been well documented [30, 33]. A recent study concluded that achieving high coverage in many designs, including symmetric and even some asymmetric designs, may be at the cost of on average 5°–14° internal rotation [25].

To date, all the studies on component coverage relied on manual component implantation [20, 25, 32], thus introducing user variability and preventing their application to large datasets. An automated and rigorous assessment of contemporary designs leveraging multi-ethnic datasets is therefore desirable. Furthermore, limited information is available on the fit of the recently developed anatomic tibial component design compared to non-anatomic designs. Utilizing a multi-ethnic dataset, this study applied comprehensive and fully automated evaluations of the component fit of contemporary anatomic and non-anatomic designs against the three competing clinical objectives. It was hypothesized that anatomic tibial component design offers increased morphological fit to the proximal tibia compared to non-anatomic designs by improving both tibial coverage and the accuracy of rotational alignment.

## Materials and methods

### Bone data

**Fig. 1:**
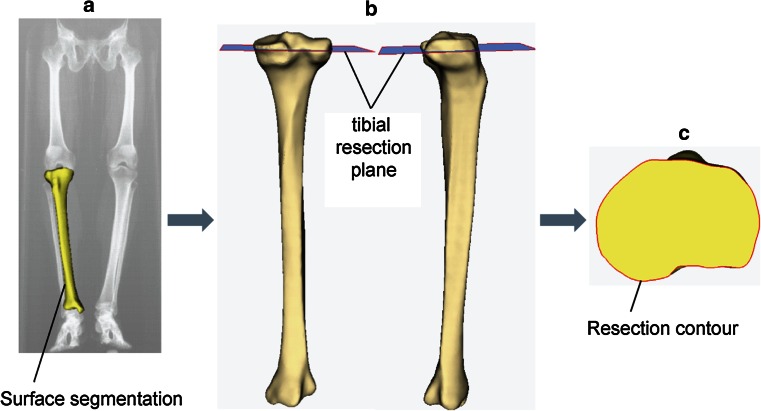
A representative tibia demonstrating the workflow for the generation of tibial TKA resection contours, consisting of **a** segmenting surface model from CT scans; **b** virtually resecting the tibia in *Zibra*; and **c** extracting resection contour from the resected bone

A total of 479 healthy right tibiae, including both Asian (*n* = 316) and Caucasian (*n* = 163) ethnicities and spanning a wide range of patient statures, were used in this study (detailed demographic information presented in Table 1). Asian subjects were live patients recruited from Indian, Chinese, Korean, and Japanese clinics following ethical approval and informed consent from each patient. CT scans of the lower extremity were performed using consistent imaging resolution (pixel size 0.75–0.85 mm, slice distance 1 mm). Caucasian data were derived from CT scans of dry bones from either the William M. Bass Donated Skeletal Collection in the Department of Anthropology or cadaver scans in the Center for Musculoskeletal Research, both at the University of Tennessee (pixel size 0.63 mm, slice distance 0.63 mm). All bone specimens were prescreened such that they had normal appearance, with no evidence of arthritic changes, prior trauma, or congenital deformities. Digital surface models (Unigraphics, Siemens PLM Software, Plano, TX, USA) of the tibiae were created through segmentation of the CT scans. A typical TKA resection was virtually performed on each tibia using *ZiBRA*™ *Anatomic Modeling System* (a proprietary software platform with advanced capabilities for digital orthopedic morphological analysis [5]), at 5°–7° posterior slope, 0° varus/valgus rotation, and 8 mm off the lateral plateau (reflecting a 10-mm surgical cut assuming a cartilage thickness of 2 mm [10]) (Fig. 1). Each resected tibia was visually approved by trained users to avoid poor quality resections. The individual tibial plateau contour following each resection was exported for further analysis (MATLAB, Mathworks, Natick, MA, USA).

**Table 1:** Demographic information on the subjects studied

Subject	Gender	*N*	Age (years, mean ± SD)	Stature (m, mean ± SD)
Indian	Male	50	53.8 ± 7.9	1.69 ± 0.06
Indian	Female	47	52.8 ± 6.9	1.55 ± 0.06
Japanese	Male	52	54.8 ± 6.0	1.67 ± 0.05
Japanese	Female	74	53.8 ± 5.6	1.54 ± 0.05
Korean*	Male	39	61.9 ± 8.3	1.69 ± 0.05
Korean*	Female	43	59.3 ± 7.4	1.55 ± 0.08
Chinese*	Male	5	70.4 ± 3.1	1.74 ± 0.04
Chinese*	Female	5	68.2 ± 3.0	1.58 ± 0.03
Caucasian	Male	98	49.7 ± 11.6	1.77 ± 0.07
Caucasian	Female	65	65.2 ± 13.5	1.61 ± 0.08

* Samples were combined into one group during analysis

### Tibial component designs

Six contemporary TKA tibial component design families were evaluated in this study (Table 2), including an anatomic Design A: *Persona*™ The Personalized Knee System (Zimmer, Warsaw, IN, USA); an asymmetric Design B: *Natural*-*Knee* ^®^ II System (Zimmer, Warsaw, IN, USA); and four symmetric designs: (1) Design C: *Vanguard* ^®^ Complete Knee System (Biomet, Warsaw, IN, USA); (2) Design D: *Triathlon* ^®^ Knee System (Stryker, Kalamazoo, MI, USA); (3) Design E: *Sigma* ^®^ Knee Solutions (Depuy Synthes, Warsaw, IN, USA); and (4) Design F: *NexGen* ^®^ Complete Knee Solution (Zimmer, Warsaw, IN, USA). All the available sizes in each component design were used in the analysis.

**Table 2:** Tibial component design families used in this study

Design	A	B	C	D	E	F
Type	Anatomic	Asymmetric	Symmetric	Symmetric	Symmetric	Symmetric
# sizes	9	7	7	8	7	10
ML size range^a^ (mm)	57.7–88.1	59.0–89.5	59.0–83.4	61.4–85.5	60.8–89.1	58.4–89.0
ML increments^a^ (mm)	3.0–5.1	5.0–5.5	3.7–4.3	3.0–5.0	2.8–6.8	0–8.0
AP increments^a^ (mm)	1.8–3.3^b^	0.5–4.6^b^	1.8–2.8	1.7–3.7	1.5–4.0	−1.5 to 4.0^c^
Genetic profile						

^a^Measured using the methods defined in the study, as demonstrated in Fig. 3a

^b^Increases asymmetrically between medial or lateral compartment

^c^Negative increment (−1.5 mm) exists only between size 8 and 9

### Component conformity to morphological metrics

**Fig. 2:**
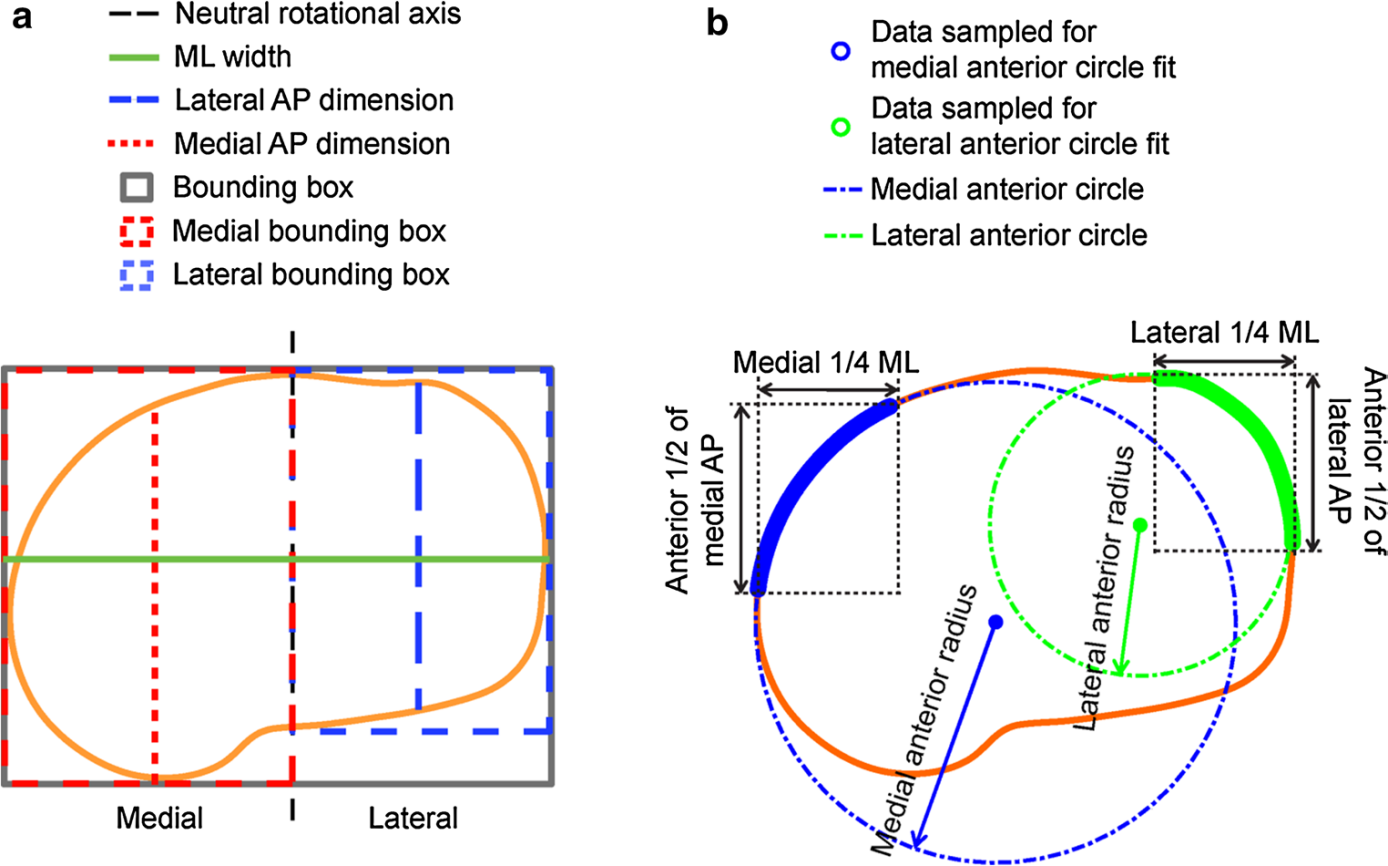
Measurements of **a** dimensions and **b** anterior radii, as adapted from Dai and Bischoff [11]

A two-dimensional coordinate system was constructed on each resected tibial surface for aligning the resection contours and performing the morphological measurements (Fig. 2). The neutral rotational axis (*Y*) was defined as the line connecting the medial third of the tubercle and the center of the posterior cruciate ligament (PCL) attachment site, projected onto the resection plane [11]. The medial and lateral compartments of the resection contours were identified as the regions separated by the neutral rotational axis. Bounding boxes were constructed in this coordinate system for the overall plateau and each compartment.

The morphology of each resection contour was quantified using a comprehensive set of morphological metrics describing *size* and *shape* (Table 3), adopted, and expanded from a recent study [11], including:

**Table 3:** Abbreviations for the morphological metrics measured

Size metric	Abbreviations	Shape metric	Abbreviations
*Dimension (mm)*		*Aspect ratio*	
Mediolateral	ML	Plateau	PAR
Medial anterior–posterior	MAP	Medial compartment	MCAR
Lateral anterior–posterior	LAP	Lateral compartment	LCAR
*Area (mm* ^*2*^ *×* *1,000)*		*Boxiness*	
Plateau	PA	Plateau	PB
Medial compartment	MCA	Medial compartment	MCB
Lateral compartment	LCA	Lateral compartment	LCB
Plateau bounding box	PBBA	*Asymmetry*	
Medial bounding box	MBBA	AP	APA
Lateral bounding box	LBBA	Anterior radius	ARA
*Radius (mm)*		Boxiness	BA
Medial anterior	MAR		
Lateral anterior	LAR		

*Dimensions* (*size*, Fig. 2a): ML width; medial and lateral AP dimensions.

*Anterior radii* (*size*, Fig. 2b): The medial profile was identified as the medial 1/4 of the resection contour; the anterior medial profile was identified as the anterior 1/2 of this medial profile. The medial anterior radius was then defined as the radius of the least squares best-fit circle to the anterior medial profile. The lateral anterior radius was defined similarly.

*Areas* (*size*): Areas enclosed by profile or bounding box (Fig. 2a) (overall and each compartment).

*Aspect ratios* (*shape*): For overall resected plateau (plateau aspect ratio), the ratio was defined as the AP/ML ratio of the bounding box. For each compartment (compartment aspect ratio), the ratio was defined as the ratio between the AP dimension of the compartment and the ML width.

*Boxiness* (*shape*): Boxiness was defined as the ratio between the area of the overall plateau or individual compartment and the area of the associated bounding box (values ranging from 0 to 1, with values closer to unity representing a boxier geometry).

*Asymmetry* (*shape*): Asymmetry metrics reflect the asymmetry between the medial and lateral compartments (values closer to unity representing more symmetric profiles). AP asymmetry was defined as the ratio between medial and lateral AP dimensions; anterior radius asymmetry was defined as the ratio between medial and lateral anterior radii; and boxiness asymmetry was defined as the ratio between medial and lateral compartment boxiness.

**Fig. 3:**
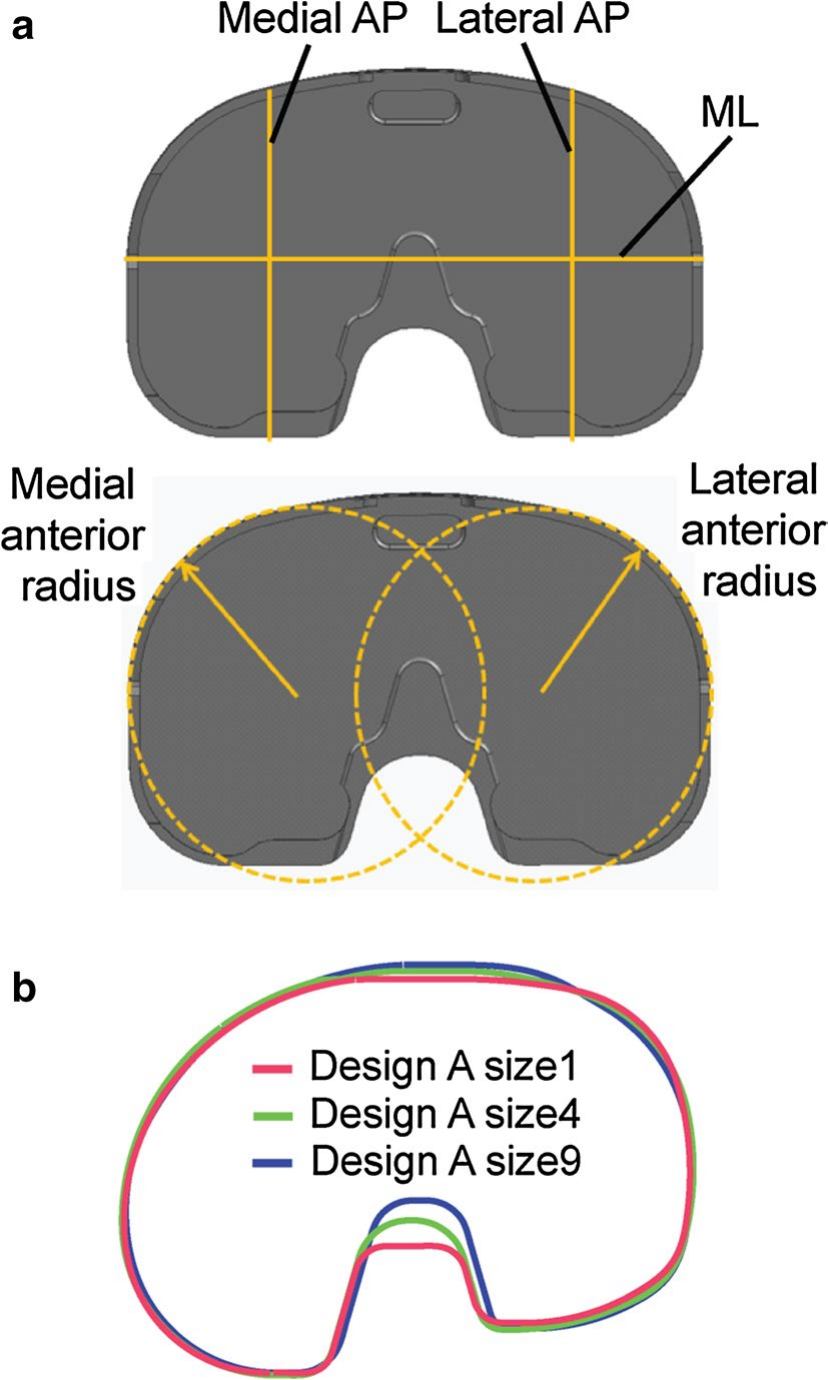
**a** Diagram showing an example of morphological measurements on the component that are similar to the measurements on the bones. **b** The profiles of smallest, median, and largest size of Design A were rescaled to match ML dimension, demonstrating the single organic shape used for the design across sizes

The metrics were regressed against ML width for each ethnicity. The regressions were compared with the same metrics measured from the six tibial component designs (Fig. 3a) to assess their morphological conformity to the resected proximal tibia.

### Component placement and fit

**Fig. 4:**
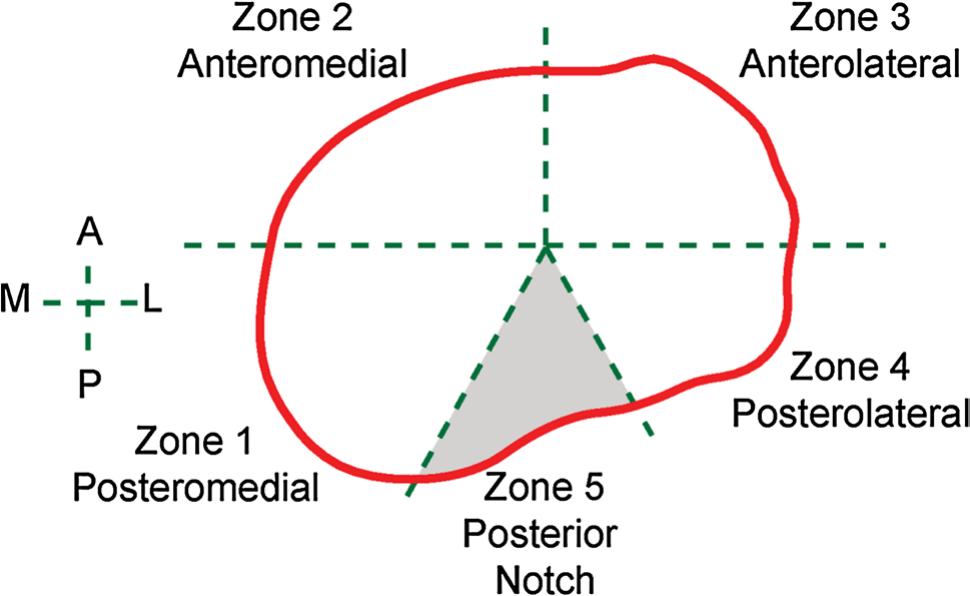
Definition of anatomic zones. The posterior notch (zone 5) was excluded from the study, as it generally corresponds to the PCL attachment and is not associated with plateau coverage

Five clinically relevant anatomic zones were identified on each resection contours as shown in Fig. 4. A fully automated algorithm was developed to virtually place the tibial components on the resected tibial surfaces. It optimized component size and placement based on all three clinically relevant objectives (rotational alignment, overhang, and coverage). Overhang and underhang were calculated as the distance from a contour point to the closest point on the component. Tibial coverage of each placement was calculated as the percentage of the resection surface covered by the tibial component (excluding zone 5, Fig. 4). The largest tibial component with the best alignment and minimal overhang was identified as the final component size and placement.

**Fig. 5:**
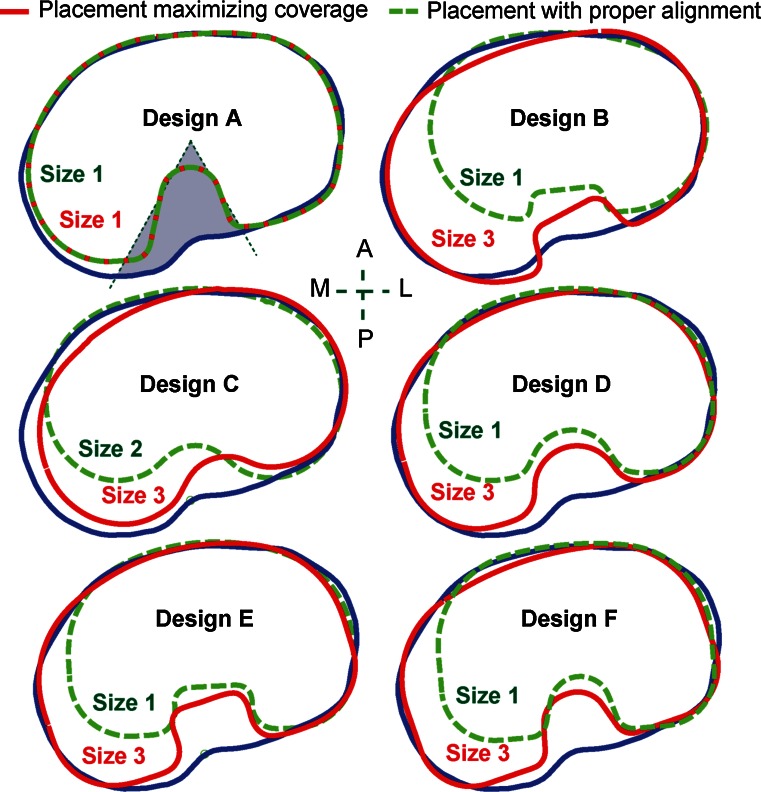
A representative tibia with the two types of component placement for the six contemporary tibial component designs

Using the automated algorithm, the next two studies applied one or both of the two types of placement described below (Fig. 5):

*Placement maximizing coverage* The largest component size was selected to maximize coverage without constraining rotation, provided ≤1 mm component overhang was achieved in zones 1–4.

*Placement with proper rotational alignment* This placement repeated the procedure as in the placement maximizing coverage, but with the added constraint that rotational alignment had to be within ±5° of the neutral rotational alignment axis.

### Ethnic/size variability in coverage

Tibial coverage from placement with proper rotational alignment was correlated with tibial component size (ML width) for each design and compared between Asian and Caucasian population groups. For cases in which a compromise on either rotation or overhang was required (due to a smaller-sized component not being available), the component design family was identified as “no suitable tibial component fit” for that bone.

### Incidence and severity of compromises during component placement

Following the above methods, downsizing of the tibial component was identified if the predicted component size when rotational accuracy (placement with proper rotational alignment) was enforced was smaller than the size which provided maximum coverage (placement maximizing coverage). In order to assess the impact of downsizing on cortical support for the tibial component, the distance from the profile of the component to the exterior cortex in anatomic zones 1–4 was calculated for both placements. Specifically, twenty points were evenly sampled on the component profile from the region that fell in each anatomic zone. The shortest distance from the sampled point to the resection contour was calculated through projecting the point to the resection contour, which represent the profile of the exterior cortex of the bone. The cortical distances for each sampled profile point were then averaged across all the bones, with smaller averages indicating better cortical support around that point on the component. The degree of mal-alignment for placement maximizing coverage and the incidence of downsizing were compared across designs.

### Institutional review board approval

The Asian CT scans in this study were collected from live patients. Each CT data collection has been approved by the institution to which the study principle investigators were primarily affiliated. The following listed the names of the institutions that granted the approval:

1. *Indian CT data* Sant Parmanand Hospital, New Delhi, India.
2. *Japanese CT data* PS Clinic, Fukuoka, Japan.
3. *Korean CT data* Department of Radiology, Asan Medical Center, Seoul, South Korea.
4. *Chinese CT data* The Affiliated Hospital of Medial College Qingdao University, Qingdao, Shandong, China.

### Statistical analysis

A power analysis was performed for the sample size selection (Minitab, Minitab Inc., State College, PA, USA). Assuming a 3 % standard deviations in tibial coverage [25], the smallest sample size in this study (*n* = 92, Korean/Chinese group) can provide a power of 0.80 to detect a 1 % difference in coverage between two groups. Therefore, the sample size used was determined to be sufficient as sub-percentage difference in tibial coverage was deemed to be clinical irrelevant.

The arithmetic mean and standard deviation of the coverage measurements were determined. One-way analyses of variance (ANOVAs) were performed to compare the tibial coverage from placement with proper rotational alignment between ethnicities and designs, and between the two types of placement across designs. Distribution of the distance from the profile of the component to the exterior cortex in each anatomic zone was calculated and compared between the two placements for each design and across designs. The null hypothesis was that all the ethnic, design, or placement group means are equal; the level of significance was defined at *p* = 0.05.

## Results

### Component conformity to morphometrics

**Fig. 6:**
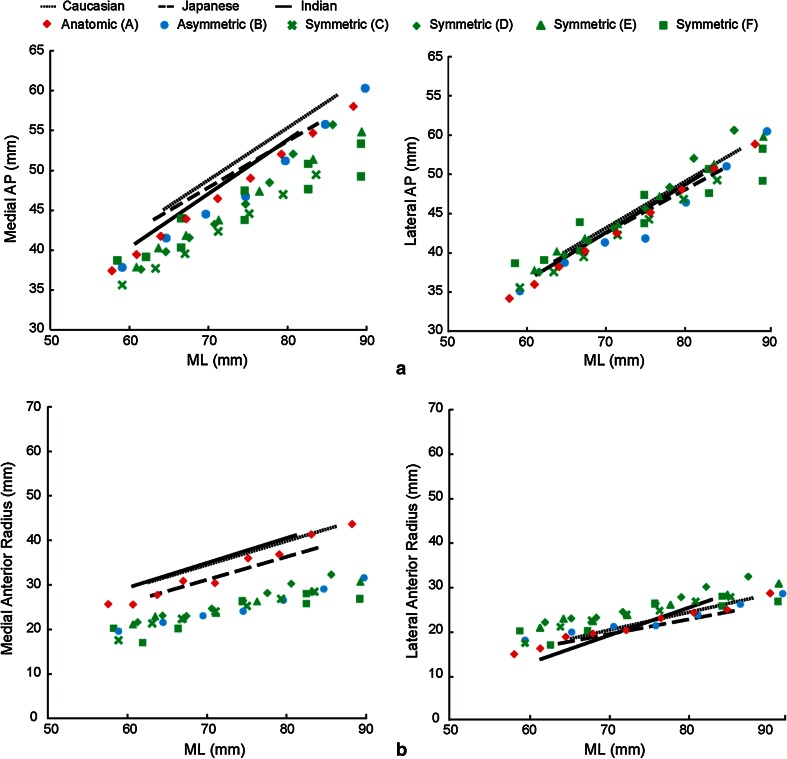
Correlations between **a** AP dimensions and **b** anterior radii with ML for each ethnicity, superimposed with associated metrics from the six contemporary tibial component designs

For *size*, the LAP of all six designs generally agreed well with the three ethnicities investigated (Fig. 6a). The anatomic design (Design A) and asymmetric design (Design B) had MAP closer to tibial anatomy, while those of the symmetric designs were smaller than the anthropometric measurement, especially for larger bones (Fig. 6a). Both tibial MAR and LAR positively correlated with ML across ethnicities (Fig. 6b), which was reflected in each design. Compared to the anatomy, both the asymmetric (B) and symmetric (C–F) designs had smaller MAR, and the symmetric designs had bigger LAR. The anatomic design (A) had the closest AP dimensions and anterior radii to the anatomy in all three ethnicities. All the area measurements on the designs were smaller than those of the tibial anatomy, with the anatomic design being closer to the natural tibia and larger than the non-anatomic designs in PBBA and MBBA. All six designs had similar values in all the other area measurements (PA, MCA, LCA, and LBBA).

**Fig. 7:**
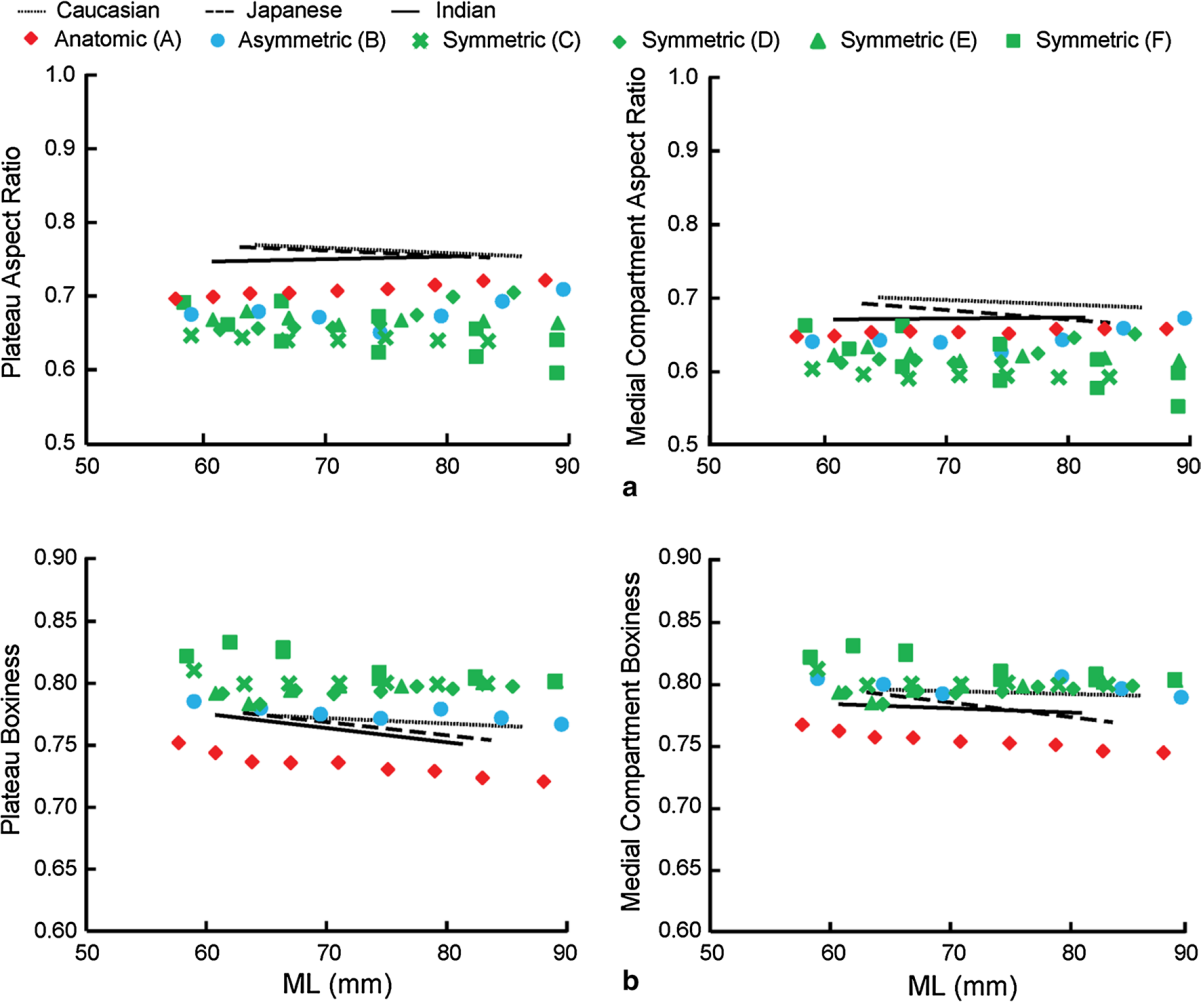
Correlations between **a** plateau and medial compartment aspect ratio and **b** plateau and medial compartment boxiness with ML for each ethnicity, superimposed with associated metrics from the six contemporary tibial component designs

**Fig. 8:**
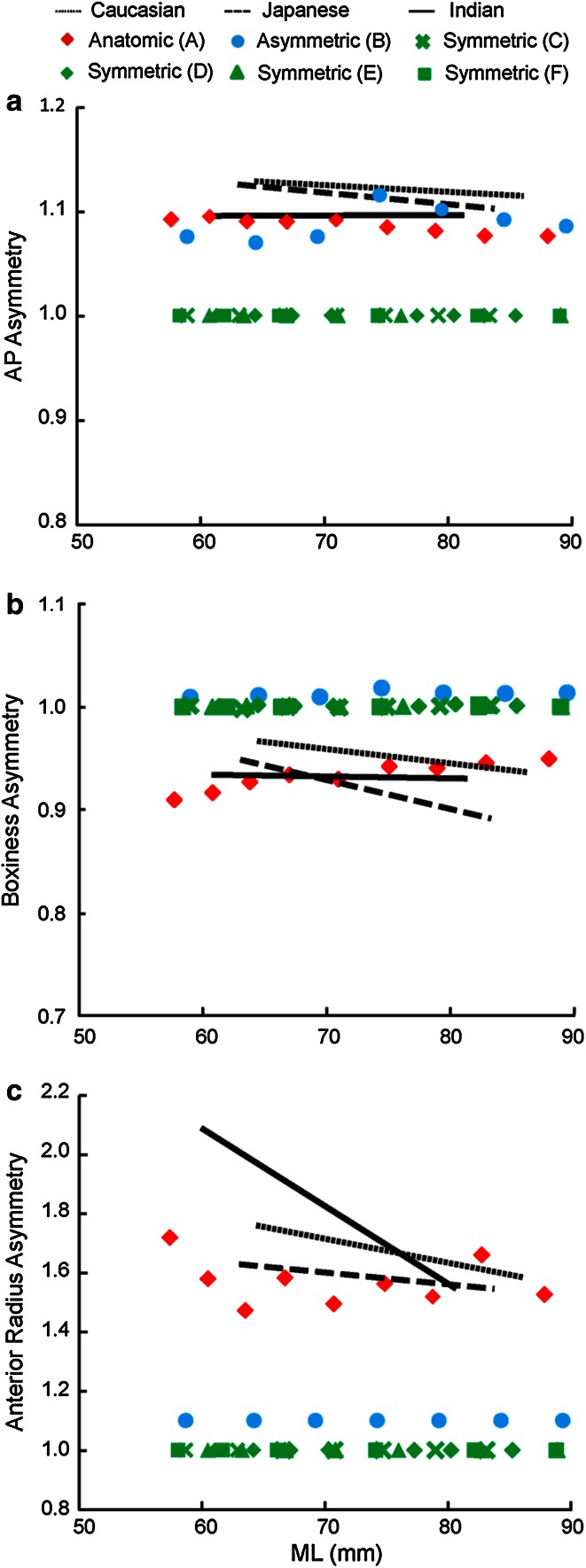
Regression between **a** AP asymmetry, **b** boxiness asymmetry, and **c** anterior radius asymmetry with ML for each ethnicity, superimposed with associated metrics from the six contemporary tibial component designs

For *shape*, the aspect ratios (PAR, MCAR, and LCAR) of the anatomic design were consistently closer to the anatomy than the other designs (PAR and MCAR shown in Fig. 7a). Both the asymmetric and symmetric designs had higher boxiness in overall plateau (PB) and medial compartment (MCB), while the anatomic design had lower boxiness measurements than the anatomy (PB and MCB shown in Fig. 7b). Both the anatomic and asymmetric designs were closer to the tibial AP asymmetry than symmetric designs (Fig. 8a). All the non-anatomic designs were symmetric or near symmetric in boxiness (BA = 1 for symmetric designs, and slightly over 1 for the asymmetric design), while a small degree of asymmetry was present in the anatomic design (BA = 0.90–0.95) (Fig. 8b). Except for the anatomic design, which closely matched the tibial anatomy, all the non-anatomic designs had constant and significantly lower anterior radius asymmetry (1 for the symmetric designs, 1.1 for the asymmetric design) (Fig. 8c).

### Ethnic and size variability in coverage

**Fig. 9:**
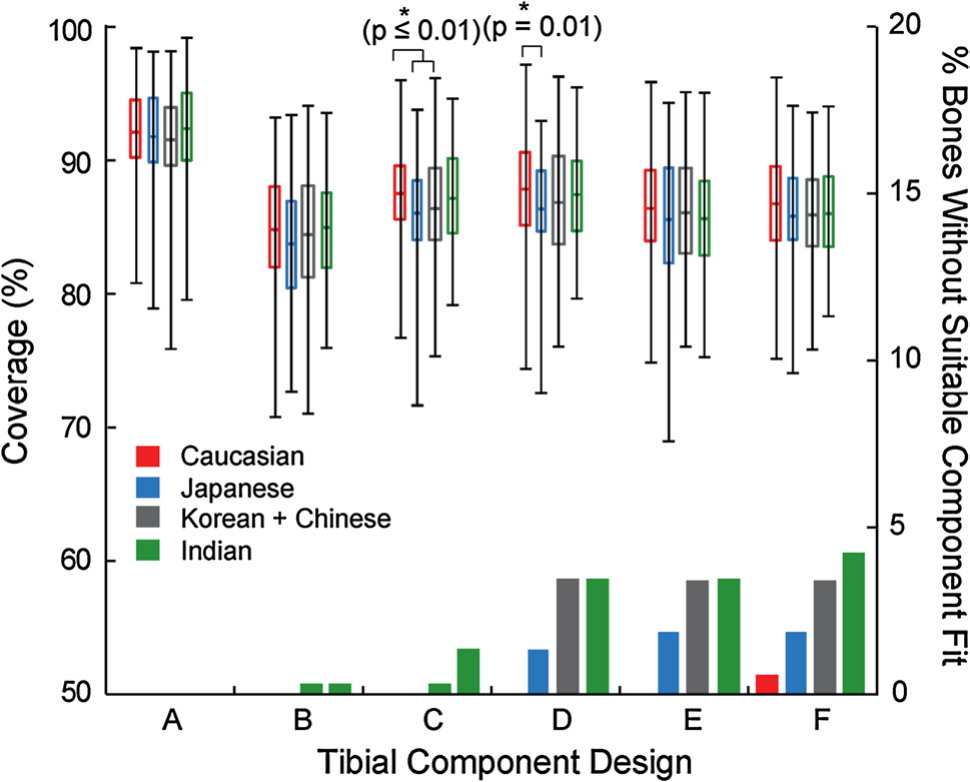
Coverage and percentage of bones without suitable component fit (due to a smaller-sized component not being available) per ethnicity for the six contemporary tibial component designs

**Fig. 10:**
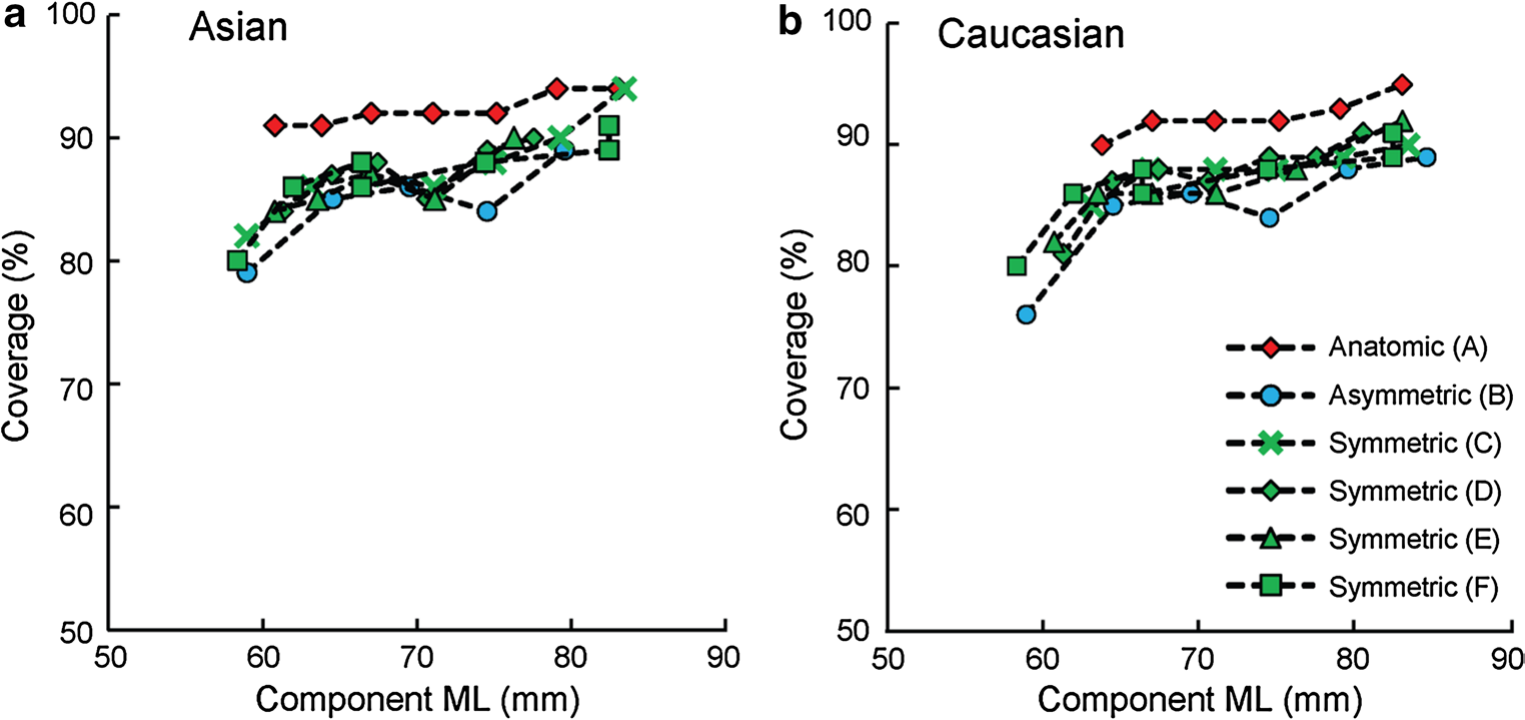
Average coverage per component size (ML width) for the six contemporary tibial component designs

With proper rotational alignment, coverage across designs and ethnicities varied (69–99 %, Fig. 9). Design A exhibited higher and more consistent average coverage (92 %) than other designs in all ethnicities (85–87 %) (*p* < 0.01). Most bones without suitable component fit were Asian (1–5 % bones for Designs B–F) and only presented in Design F for Caucasian (1 %) (Fig. 9). Significantly (<1.5 %) higher coverage was found for Caucasians compared to Korean and Chinese in Design D (*p* = 0.01) and to Japanese in Designs C and D (*p* ≤ 0.01) (Fig. 9). No differences were found within Asian ethnicities in coverage. Coverage generally decreased with reduced component ML (Fig. 10), with the differences in coverage between the largest and smallest component ML ranging from 3 % (Design A in Asian) to 13 % (Design B in Caucasian).

### Incidence and severity of compromises during component placement

**Fig. 11:**
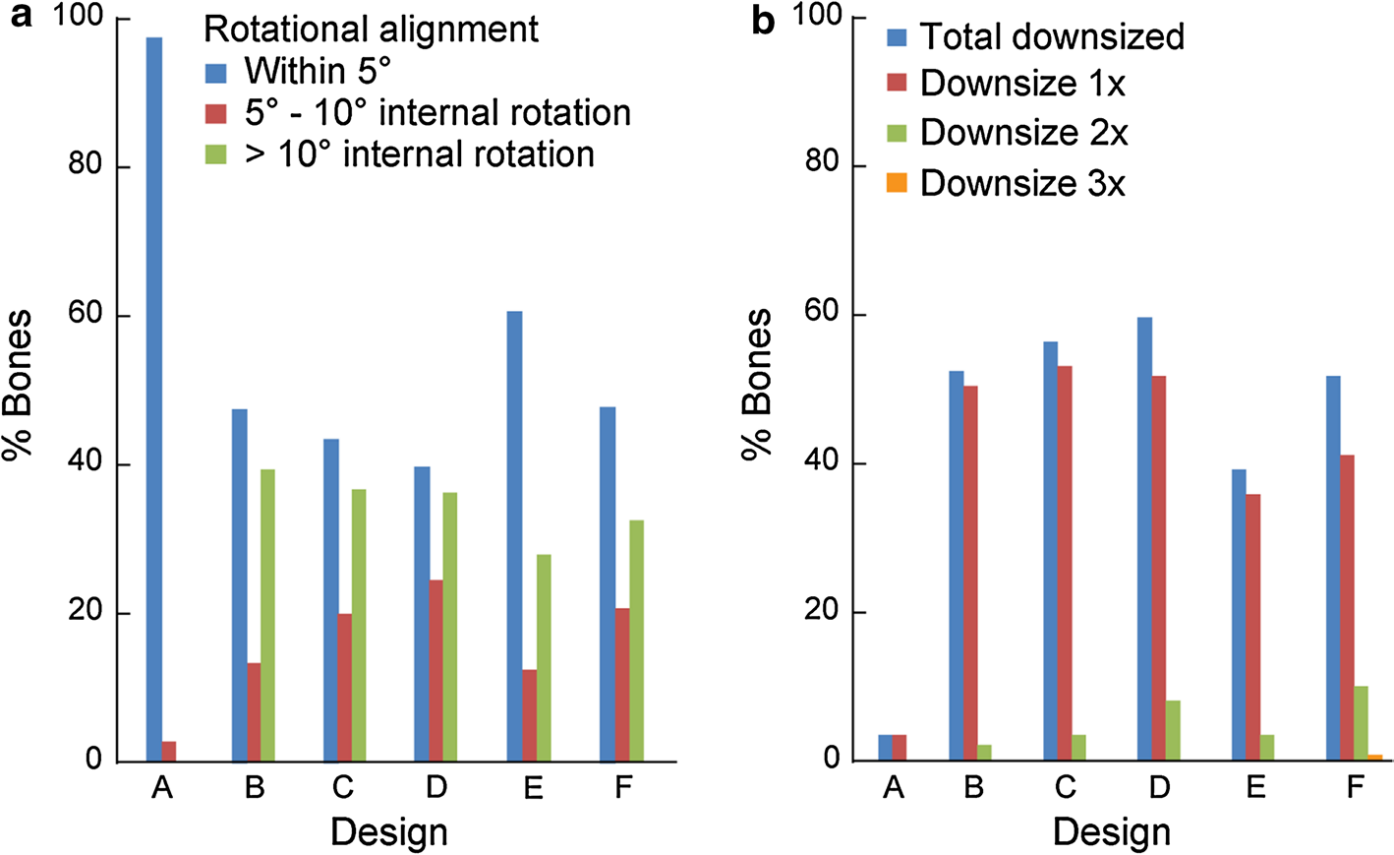
**a** Percentages of bones that require component to be internally rotated to maximize coverage. **b** Percentage of bones with tibial component downsized, and extent of downsizing

Component internal rotation of more than 5° was required on 39–60 % of bones in the non-anatomic designs in order to maximize coverage, leading to component downsizing (sometimes multiple times). Furthermore, 30 % of the bones were internally rotated beyond 10°, and 2–11 % of the bones required downsizing of 2 or more sizes. In contrast for the anatomic design, only 3 % of the bones required a single downsize caused by small mal-rotations (≤10°) (Fig. 11).

**Fig. 12:**
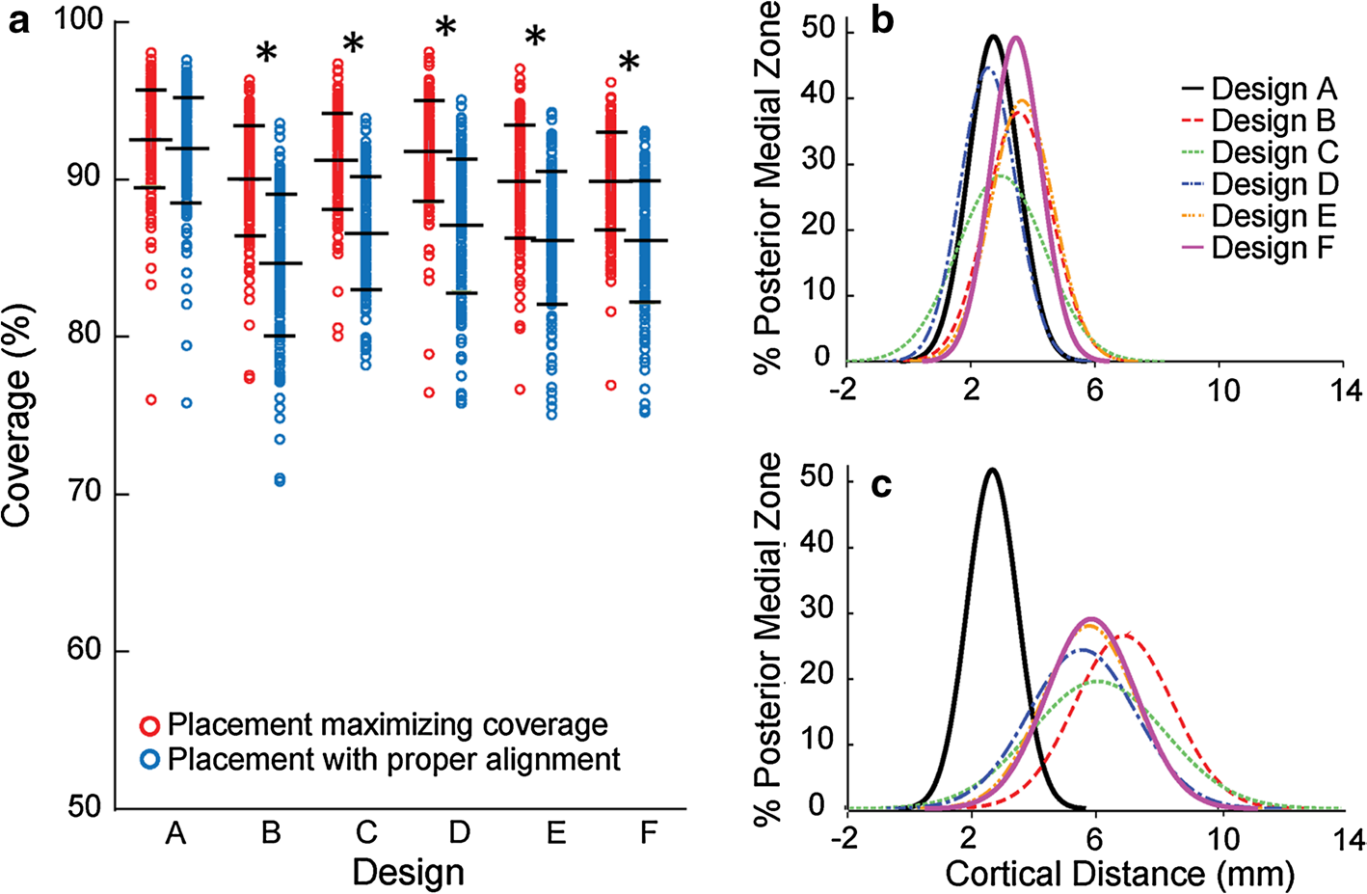
**a** Coverage per design (mean and std. dev. indicated). *Statistical difference between placement maximizing coverage and placement with proper alignment (*p* < 0.01).* Histogram* of the average distance from tibial tray to exterior cortex in the posterior medial region for **b** placement maximizing coverage and **c** placement with proper alignment

Across designs, in order to ensure proper rotational alignment, average coverage decreased from 90–93 to 85–92 % (Figs. 5, 12a). In the non-anatomic designs (B–F), enforcing proper alignment significantly compromised coverage (up to 20 %, on average 4–6 %, *p* < 0.01) (Fig. 12a) and posteromedial cortical support (zone 1, from 2.6–3.6 mm on average to 5.6–7.0 mm on average, *p* < 0.01) (Fig. 12b, c). In contrast, the anatomic design (A) decreased less than 0.5 % in coverage and had negligible change (<0.01 mm) in posteromedial cortical support, with higher coverage in both placements and better posteromedial cortical support with proper alignment than the other designs (*p* ≤ 0.03). No differences in cortical support were found in other anatomic zones (2–4) between the two placements (n.s.).

## Discussion

The most important finding of the present study is that the anatomic tibial component design offers improved component fit compared to the non-anatomic designs investigated. It exhibited the closest morphological match to the size and shape of the resected tibia, provided the highest and most consistent tibial coverage, and resulted in the least compromise across the multi-ethnic dataset studied. Specifically, for the anatomic design, by properly leveraging anatomic data within component design, the accuracy of rotational alignment is facilitated, not impeded, by maximizing tibial coverage and minimizing overhang. In contrast, the surgical compromise required for the non-anatomic designs may be attributed primarily to the morphological mismatches between their profiles and the resected tibia, such as smaller medial compartment and overlooked or insufficient asymmetry in the anterior radius. To ensure accurate alignment, symmetric designs that have a smaller medial compartment have an increased tendency to overhang in the posterolateral region of the resected tibia [17]. Mismatch in medial compartment size and anterior radii requires tray malrotation to maximize coverage or forces a compromise on coverage posteromedially in order to preserve proper rotation. In contrast, the profile of the anatomic design grows progressively around a single anatomic shape (Fig. 3b) and reflects all three definitions in asymmetry (AP, boxiness, and anterior radius) in the resected tibia. Adequate AP asymmetry and boxiness asymmetry provide increased tibial coverage, while proper asymmetry in the anterior radius provides a means to guide accurate rotational alignment through matching the medial and lateral anterior radii of the component with those of the resected tibia during placement. As a result, the anatomic design has the highest and most consistent coverage (<3 % variation) across all ethnicities and size ranges investigated (Fig. 10) and is most resistant to mal-rotation (Fig. 11), consistent with previous studies on anatomic designs [18, 25].

It is worth noting that for the non-anatomic designs, although smaller (ML) component sizes were used for the dataset compared to those for the anatomic design (Fig. 10), there still were 1–5 % of bones that could not be fit without incurring significant component overhang under proper rotational alignment (Fig. 9). These data agree with observations of increased tibial component mismatch for Asian populations [9, 19, 34, 37] and further confirm that smaller sizes of the non-anatomic designs do not fully reflect small bone anatomy, thus forcing a compromise in one or more placement objectives. Also, consistent with previous results which showed that the majority of the morphological variability can be attributed to size, not intrinsic shape differences between ethnicities [11], no clinically meaningful differences in coverage were observed between ethnicities in this study.

There are several limitations to this study. First, although the resection parameters used for design comparison were representative of a typical TKA resection, design-specific tibial resections were not investigated. Second, ideal tibial resection was employed (e.g., only 5°–7° posterior slopes were investigated); however, clinical variability in resection parameters has been reported in previous studies [26, 28]. Third, this study did not considered the variability between different anatomic rotational alignment axes [29]. Finally, all the results here were based on healthy subjects, not TKA candidates. The impact of these limitations on the results may require further investigation.

Although all the measurements performed in this study were based on fully automated computer simulation, the expected resolution of the results is impacted by several aspects of the data pre-processing: (1) Accuracy of the automatic annotations of the landmarks depends on the resolution of the CT data, which had sub-millimeter accuracy (up to 2 decimal places); (2) approval of automatically defined landmarks by experienced users introduces some level of inter- and intra-user variability, though this has been shown to be at sub-millimeter level (errors reported in 1 decimal place) [35]; and (3) the surgical reference axes for the resection were constructed based on anatomic landmarks and naturally inherited the errors in landmark identification. Based on these considerations, results here were reported at a comparable level of resolution. Additionally, the accumulated impact of variability from CT data on morphometric analysis of tibial resections has been shown to be within typical clinical bounds of TKA for the workflow utilized here [12], which supports the clinical relevancy of the virtual tibial resection in this study.

Good morphological fit between the tibial component and the resected tibial anatomy is an important factor for long-term success in TKA. This study comprehensively evaluated contemporary tibial component designs. Here, the term “good fit” not only means a close match in the basic dimensions or aspect ratios with the resected tibia, but also the design appropriately accommodates balancing of tibial coverage with accuracy in rotational alignment for proper kinematics and minimal overhang to avoid soft tissue impingement. The clinical implications of the observations in the present study revealed that the anatomic design allows for increased accuracy in rotational alignment of the tibial component with better proximal tibial coverage. Accurate rotational alignment of the tibial component improves knee kinematics and patella tracking, while better proximal tibial coverage influences fixation and reduces peripheral soft tissue impingement. These features influence the ultimate clinical outcome of TKA. The results here further revealed the current gap in the morphological fit of symmetric and asymmetric tibial component designs, demonstrating that one or more of the key placement objectives (coverage, rotation, and overhang) often need to be compromised in many contemporary designs.

## Conclusion

Assessment of contemporary tibial component profiles using morphological measurements as well as simulated component sizing and placement indicates that anatomic designs most effectively balance the competing surgical goals of tibial coverage, proper rotation, and minimal component overhang.
